# Combined elevation of TRIB2 and MAP3K1 indicates poor prognosis and chemoresistance to temozolomide in glioblastoma

**DOI:** 10.1111/cns.13197

**Published:** 2019-07-18

**Authors:** Jia Wang, Jie Zuo, Alafate Wahafu, Mao‐de Wang, Rui‐chun Li, Wan‐fu Xie

**Affiliations:** ^1^ Department of Neurosurgery The First Affiliated Hospital of Xi'an Jiaotong University Xi'an China; ^2^ Center of Brain Science The First Affiliated Hospital of Xi'an Jiaotong University Xi'an China; ^3^ The Second Affiliated Hospital of Xi'an Jiaotong University Xi'an China

**Keywords:** glioblastoma, MAP3K1, prognosis, therapy resistance, TRIB2

## Abstract

**Introduction:**

Glioblastoma (GBM) is the most lethal primary malignant brain tumor in adults with poor survival due to acquired therapeutic resistance and rapid recurrence. Currently, the standard clinical strategy for glioma includes maximum surgical resection, radiotherapy, and temozolomide (TMZ) chemotherapy; however, the median survival of patients with GBM remains poor despite these comprehensive therapies. Therefore, the identification of new prognostic biomarkers is urgently needed to evaluate the malignancy and long‐term outcome of glioma.

**Aims:**

To further investigate prognostic biomarkers and potential therapeutic targets for GBM.

**Results:**

In this study, we identified tribbles pseudokinase 2 (TRIB2) as one of the genes that is most correlated with pathological classification, radioresistance, and TMZ resistance in glioma. Additionally, the expression of mitogen‐activated protein kinase kinase kinase 1 (MAP3K1) showed a strong correlation with TRIB2. Moreover, a combined increase in TRIB2 and MAP3K1 was observed in GBM and indicated a poor prognosis of patients with glioma. Finally, enriched TRIB2 expression and MAP3K1 expression were shown to be associated with resistance to TMZ and radiotherapy.

**Conclusion:**

Combined elevation of TRIB2 and MAP3K1 could be novel prognostic biomarkers and potential therapeutic targets to evaluate the malignancy and long‐term outcomes of GBM.

## 1 INTRODUCTION

Glioblastoma (GBM) is one of the most lethal and prevalent tumors in the adult central nervous system, with a morbidity of approximately 7/10 000.^1^ Currently, the standard clinical strategy for glioma includes maximum surgical resection, radiotherapy, and temozolomide (TMZ) chemotherapy. However, the median survival for patients with GBM remains only 14.6 months.^1^ Based on the pathological characteristics, glioma can be categorized into four grades (from I to IV), which are widely used to assess the outcomes of patients with glioma.^2^ Increasing evidence has indicated that the traditional classification of glioma provides very little insight into the biological behavior and molecular mechanisms that could be used as therapeutic targets. Therefore, the identification of new prognostic biomarkers is urgently needed to evaluate the malignancy and long‐term outcome of glioma.^3^, ^4^

Tribbles pseudokinase 2 (TRIB2) is a kinase‐encoding gene of the tribbles family. TRIB2 was first identified to regulate mitosis and embryonic development in Drosophila.^5^ Recent studies have indicated that TRIB2 functions as a crucial oncogene that regulates a wide range of cellular processes, including tumorigenesis, proliferation, invasion, and therapeutic resistance, in various subtypes of cancer, such as small cell lung cancer, liver cancers, colorectal cancer, and acute myeloid leukemia.^5^, ^6^, ^7^, ^8^ Foulkes et al^9^ demonstrated that TRIB2 expression is significantly increased in acute myeloid leukemia and promotes tumor proliferation by inactivating CCAAT‐enhancer‐binding protein α (C/EBPα). Moreover, Wnt/TCF signaling‐dependent activation of TRIB2 stimulates the proliferation of liver cancer cells through a stabilization effect on yes‐associated protein (YAP).^10^ Hou et al^7^ found that TRIB2 increases colorectal cancer tumor growth by decreasing the expression of p21, which functions as a tumor suppressor gene, indicating that TRIB2 could become a potential molecular target for colorectal cancer. These studies prompted the hypothesis that TRIB2 is a prognostic predictor for multiple types of malignant tumors. However, the functional role of TRIB2 and the underlying mechanism in glioma are poorly elucidated.

Mitogen‐activated protein kinases (MAPKs) are essential regulators of evolutionarily conserved proteins that mediate various cellular physiologies.^11^ The activation of MAPK networks includes 3 sequential phosphorylation strategies, from MAPK kinase kinases (MAP3Ks) to MAPK kinase (MAP2Ks) and MAPKs.^12^ Mitogen‐activated protein kinase kinase kinase 1 (MAP3K1), which is an important isoform for the first stimulation step of MAPKs, participates in regulating physiological and pathological processes, such as cell growth, cell migration, and apoptosis in gastric cancer, breast cancer, and nonsmall cell lung cancer cells.^13^, ^14^, ^15^ Additionally, a mechanistic study has indicated that MAP3K1 promotes tumor progression via the specific phosphorylation of MAP2K4 and thus selectively phosphorylates and activates c‐Jun N‐terminal kinase (JNK).^16^ MAP3K1 also activates JNK through the phosphorylation of MAP2K1/2 and MAP2K716. Despite these results, the function and mechanism of MAP3K in glioma are still not fully understood.

Increasing data have shown that kinase‐dependent activation of tumorigenesis and therapeutic resistance is one of the major causes for GBM tumor recurrence, indicating that aberrant enhanced kinase activity could be a potential predictive biomarker for GBM.^4^, ^17^, ^18^, ^19^ In this study, to further investigate prognostic biomarkers for glioma, kinome‐wide screening was first performed with 3 GEO datasets (Mao et al,^20^ 2015, Tso et al,^21^ 2015, and Wang et al,^22^ 2017). TRIB2 and MAP3K1 were identified as genes that are correlated with the pathology and survival of glioma. Moreover, a combined increase in TRIB2 and MAP3K1 in glioma is associated with a poor prognosis and therapeutic resistance to TMZ and radiotherapy. These data suggest that TRIB2 and MAP3K1 could be used as novel therapeutic targets and prognostic biomarkers to evaluate the malignancy and long‐term outcome of GBM.

## 2 MATERIALS AND METHODS

### 2.1 Patient and glioma samples

This study was approved by the patients and the Scientific Ethics Committee at the First Affiliated Hospital of Xi'an Jiaotong University (approval no. 2016‐18). A total of 91 glioma samples and three nontumor tissue samples from epilepsy surgery were collected from patients who underwent surgical operations from 2008 to 2016. All necessary consent forms and documents were signed before the surgeries were performed. All samples were embedded in paraffin blocks, and 17 glioma samples and three nontumor tissues were immediately frozen and stored in liquid nitrogen for RNA purification.

### 2.2 Gene expression analysis

Gene expression data were extracted from GEO datasets (Mao et al,^20^ 2015, Tso et al,^21^ and Wang et al^22^). Hierarchical bi‐clustering was performed to analyze the expression of the target genes by Cluster 3.0 software. Euclidean distance and average linkage were used as a similarity metric and clustering method, respectively. Comparisons of the relative gene expression between the different groups are presented as fold changes.

### 2.3 Gene set enrichment analysis (GSEA) and Kyoto encyclopedia of genes and genomes (KEGG) analysis

Gene expression profiles were derived from public databases, including the Cancer Genome Atlas (TCGA RNA sequencing) database and Gene Expression Omnibus (GEO, GSE16011). All data were preprocessed, including normalization and gene ID transformation, by R software, and these data were then ordered by the expression level of TRIB2 to divide all samples into two groups (TRIB2^high/low^) according to the median. The data were then ordered by the expression level of MAP3K1 to generate TRIB2^High^/MAP3K1High, TRIB2^High^/MAP3K1Low, TRIB2^Low^/MAP3K1High, and TRIB2^Low^/MAP3K1Low groups. Subsequently, the limma package (PMID: 25605792) was used to identify differentially expressed genes in the TRIB2^High^/MAP3K1High and TRIB2^Low^/MAP3K1Low groups. Next, the Database for Annotation, Visualization and Integrated Discovery online tool (DAVID, https://david.ncifcrf.gov/) was utilized to conduct gene ontology (GO) annotation and KEGG pathway enrichment analysis. The results are presented as a chord and bubble diagram, respectively. Furthermore, GSEA was carried out according to User's Guide (http://software.broadinstitute.org/cancer/software/gsea/wiki/index.php) to elucidate crucial pathways that are correlated with TRIB2^High^/MAP3K1High and TRIB2^Low^/MAP3K1Low (PMID: 16199517).

### 2.4 Quantitative RT‐PCR (qRT‐PCR)

Total RNA was prepared using an RNeasy mini kit according to the manufacturer's instructions. The RNA concentration was determined by a NanoDrop 2000 instrument, and cDNA was synthesized using iScript reverse transcription5 supermix for qRT‐PCR according to the manufacturer's protocol. qRT‐PCR was performed by using a StepOnePlus real‐time PCR system with SYBR Select Master Mix (Applied Biosystems). GAPDH was used as an internal control. The cycles for DNA amplification were as follows: 94°C for 2 minutes, 50 cycles at 94°C (30 seconds), 60°C (30 seconds), and 72°C (40 seconds). The sequences of the primers are as follows: TRIB2 forward: ATGAACATACACAGGTCTACCCC; TRIB2 reverse: GGGCTGAAACTCTGGCTGG; MAP3K1 forward: CATCAGGTCGCACA GTGAAAT; MAP3K1 reverse: TCAGGGCTATATGGTGAGAAGC; GAPDH forward: GAAGGTGAAGGTCGGAGTCA; and GAPDH reverse: TTGAGGTCAATGAAGGGGTC. Relative quantification of cDNAs to GAPDH was conducted by the 2^−ΔΔCt^ method.

### 2.5 Immunohistochemistry (IHC)

IHC was performed as previously described.^4^, ^23^ Anti‐TRIB2 and anti‐ MAP3K1 primary antibodies were purchased from Invitrogen (catalog no. MA5‐24942). Horseradish peroxidase‐conjugated goat anti‐rabbit IgG (catalog no. ab97051) and goat anti‐mouse IgG were obtained from Abcam (catalog no. ab205719) and used as secondary antibodies. All glioma samples used in this study were pathologically diagnosed, and recurrence was confirmed by computed tomography (CT) or magnetic resonance imaging (MRI). Nuclei were counterstained with hematoxylin.

### 2.6 German immunohistochemistry score (GIS)

GIS was used to analyze the expression of TRIB2 and MAP3K1 as previously described.^22^ The immunoreactivity score was calculated according to the following equation: immunoreactivity score = positive cell score × staining intensity score. The positive cell score was scored as follows: 0, negative; 1, <10% positive; 2, 11%‐50% positive; 3, 51%‐80% positive; and 4, >80% positive. The staining intensity score was scored as follows: 0, negative; 1, weakly positive; 2, moderately positive; and 3, strongly positive. An immunoreactivity score >5 was considered high expression, and ≤5 was defined as low expression.

### 2.7 Receiver operating characteristic (ROC) analysis

For ROC analysis, all patients were divided into two groups based on the survival period. The expression of the genes of interest was calculated by GIS, and ROC analysis was performed with SPSS 22.0 software. The area under the curve (AUC) was used to evaluate the specificity and sensitivity of each cutoff. The statistical significance was compared with a *Z* test.

### 2.8 Statistical analysis

All the results in this study are presented as the mean ± standard deviation. The number of replicates is mentioned in the related figure legends. Statistical differences between two groups were evaluated using 2‐tailed *t* tests. Multiple groups were compared with one‐way ANOVA followed by Dunnett's posttest. Kaplan‐Meier survival analysis was compared by the log‐rank test. All statistical analyses were performed with SPSS 22.0 or GraphPad Prism 6 software. A *P*‐value less than 0.05 indicated statistical significance.

## 3 RESULTS

### 3.1 Kinome‐wide screening for prognostic biomarkers in glioma

**Figure 1:**
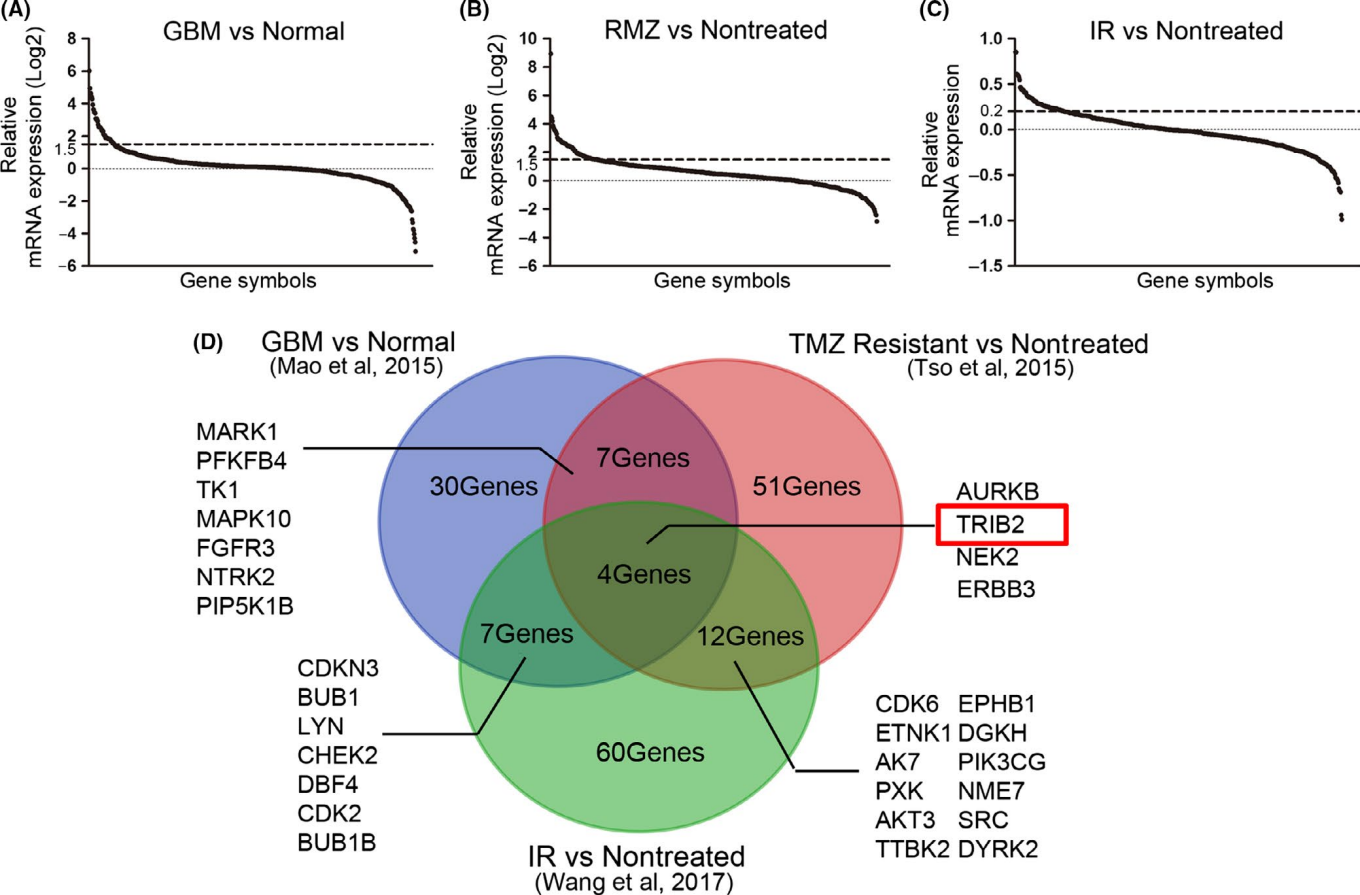
Kinome‐wide screening identified TRIB2 as a prognostic biomarker in glioma. A, Kinome‐wide microarray analysis for 668 kinase‐encoding genes in GBM neurosphere compared with the nontumor (Mao et al,^20^ 2015). B, Kinome‐wide microarray analysis for 668 kinase‐encoding genes in TMZ‐resistant glioma cell lines compared with the nontreated cells (Tso et al,^21^ 2015). C, Kinome‐wide microarray analysis for 668 kinase‐encoding genes in radiation‐treated glioma cell lines (IR) compared with the nontreated cells (Wang et al,^22^ 2017). D, Venn diagram indicated that TRIB2 was one of the four most differentially expressed kinase‐encoding genes in these three databases

To thoroughly explore the potential prognostic biomarkers for glioma, kinome‐wide hierarchical bi‐clustering was performed using previously published microarray databases related to TMZ resistance, World Health Organization (WHO) grade, and radiotherapeutic resistance (Tso et al,^21^ 2015; Mao et al,^20^ 2015 and Wang et al,^22^ 2017). The differentially expressed genes were sorted by their fold changes (Figure 1A‐C, and Tables [Link], [Link], [Link]). The genes that were more than 1.5‐fold upregulated were selected from Tso et al^21^ and Mao et al^20^ Moreover, genes that were upregulated by 0.2‐fold were extracted from the Wang et al^22^ database. The overlapping genes from those three gene lists are summarized. Finally, we found that four kinase‐encoding genes were significantly correlated with characteristics of malignancy, including TMZ resistance, WHO grade, and radiotherapeutic resistance in GBM (Figure 1D). Since TRIB2 was identified as an essential oncogene in a variety of cancer types, we focused on TRIB2 for further characterization of glioma in this study.

### 3.2 MAP3K1 expression was correlated with TRIB2 in glioma

**Figure 2:**
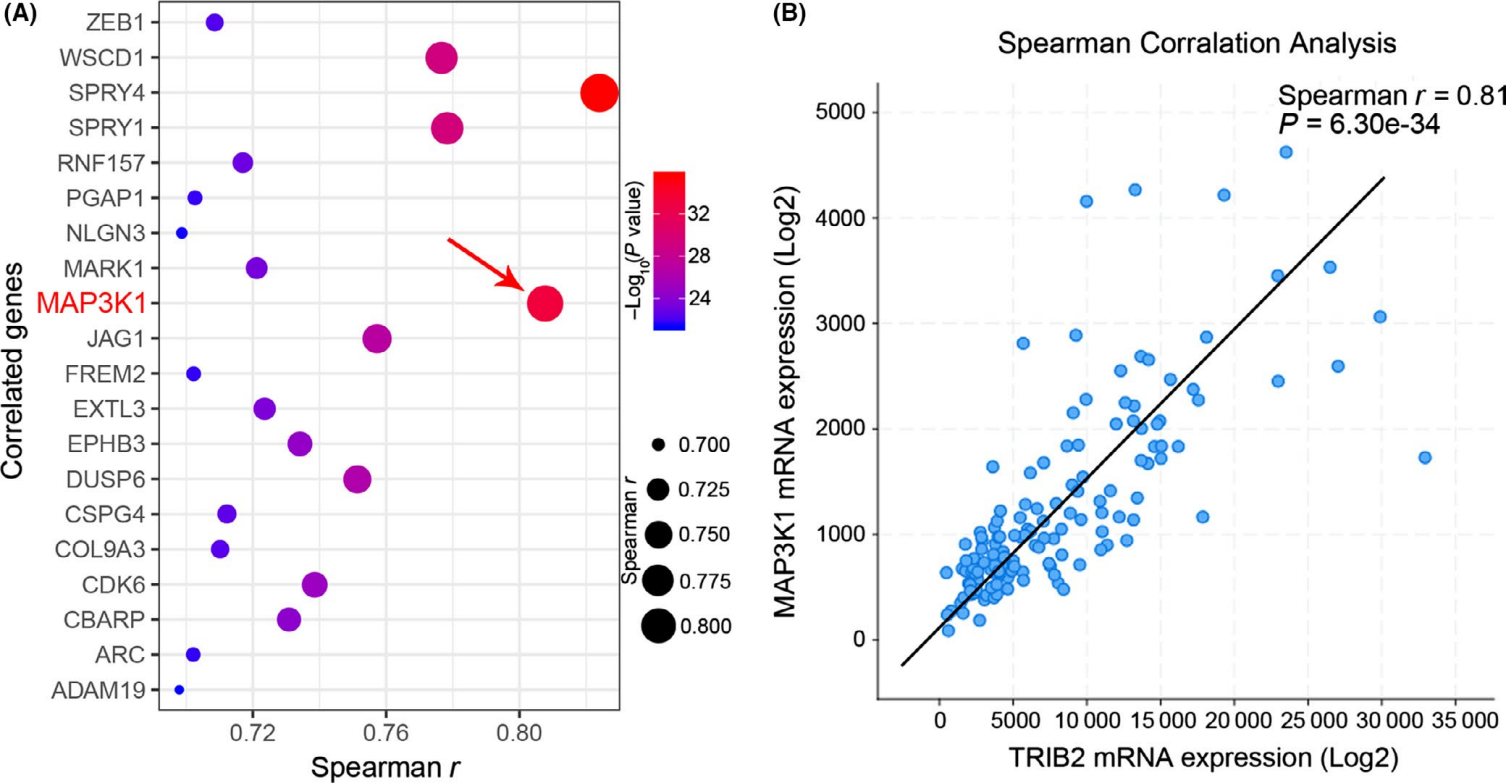
MAP3K1 expression was correlated to TRIB2 in glioma. A, Spearman correlation analysis with TCGA showed that MAP3K1 was among the most correlated genes to TRIB2. B, TRIB2 mRNA showed a strong correlation to MAP3K1 mRNA in TCGA database (Spearman *r* = 0.81, *P* < 0.01, with paired *t* test)

Next, to investigate the candidate gene correlated with TRIB2, Spearman's correlation analysis was performed using TCGA RNA sequencing database. The results demonstrated that MAP3K1 is one of the most closely associated genes with TRIB2 in glioma (Figure 2A,B), indicating that MAP3K1 might be functionally regulated by TRIB2 and could be used as a potential combined biomarker for glioma. The detailed results are shown in Table S4.

### 3.3 TRIB2 and MAP3K1 were upregulated in gliomas

**Figure 3:**
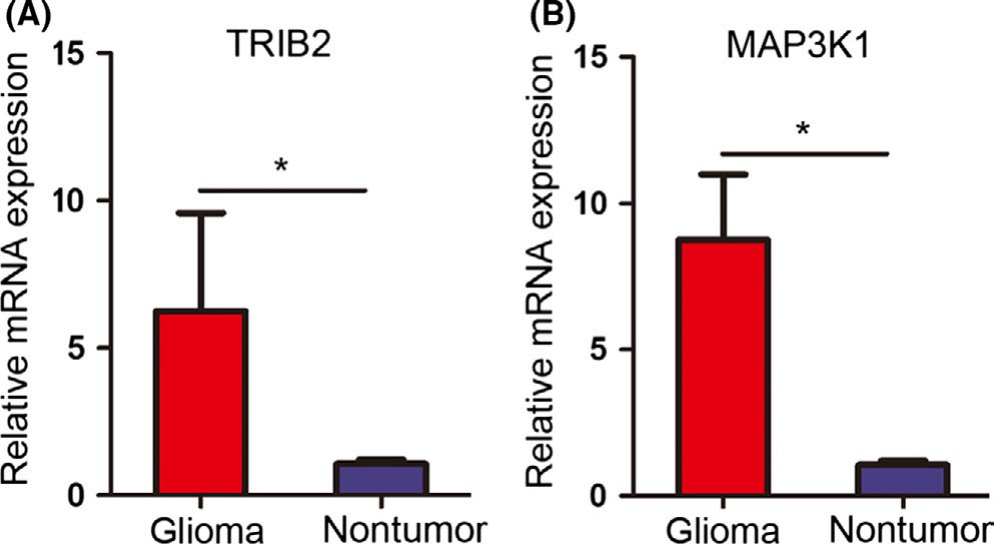
TRIB2 and MAP3K1 were upregulated in gliomas. A‐B, qRT‐PCR showed elevated TRIB2 (A) and MAP3K1 (B) expression in 17 glioma samples compared with three nontumor tissues (*P < 0,05, with *t* test)

**Figure 4:**
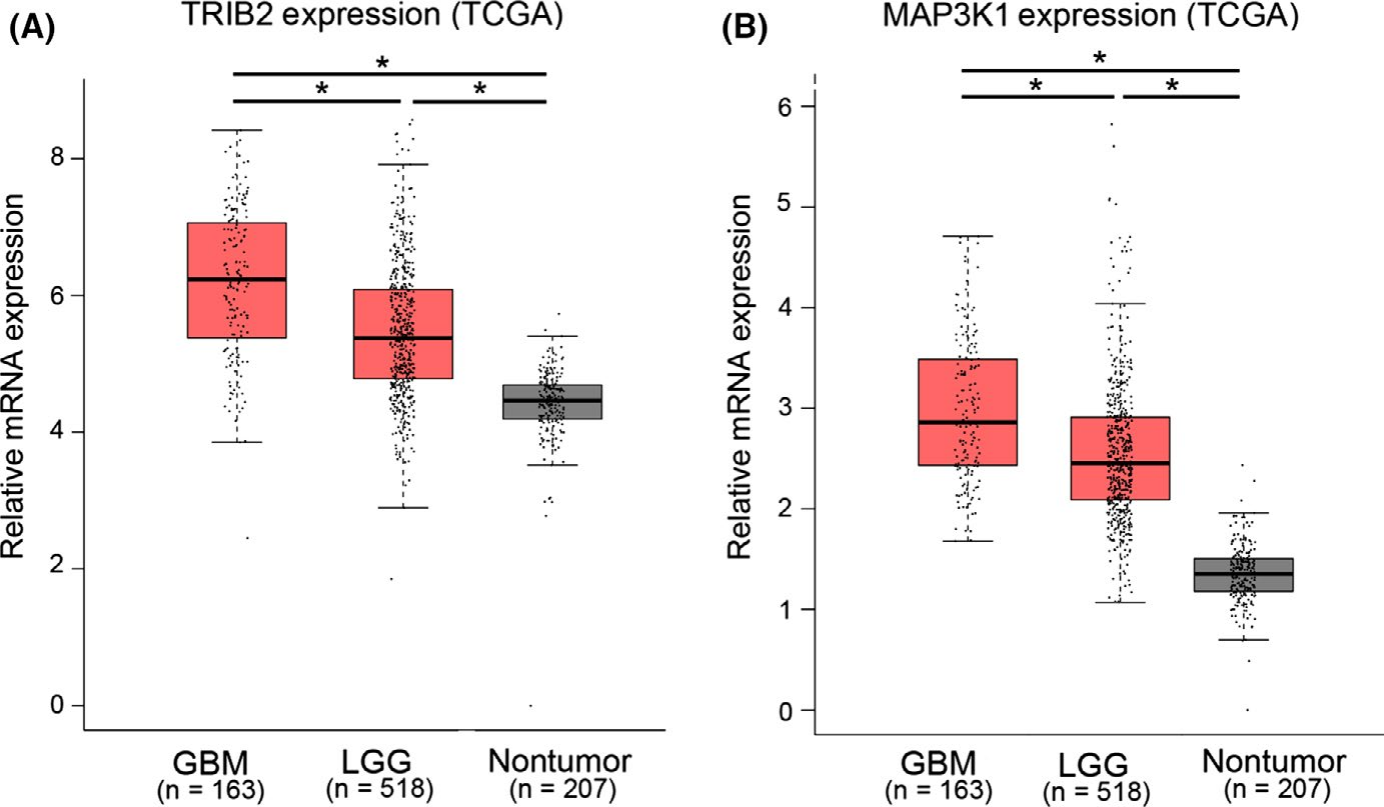
Gene expression analysis for TRIB2 and MAP3K1 in TCGA. A‐B, Gene expression analysis with TCGA database showed that TRIB2 (A) and MAP3K1 (B) were highly expressed in GBM and low‐grade glioma (LGG) when compared to nontumor tissue (*P < 0.05)

Seventeen glioma samples and three nontumor tissues were collected for RNA purification, and the expression levels of TRIB2 and MAP3K1 were evaluated by qRT‐PCR. The results showed a significant increase in TRIB2 and MAP3K1 in the glioma samples compared with the nontumor samples (Figure 3A,B). Similarly, we assessed the expression of TRIB2 and MAP3K1 in glioma/GBM by analyzing the TCGA (RNA sequencing) database, and the results demonstrated that TRIB2 and MAP3K1 were markedly increased in glioma tissues, especially GBM tissues, compared with nontumor tissues (Figure 4A,B).

### 3.4 TRIB2 and MAP3K1 were significantly enriched in GBM

**Figure 5:**
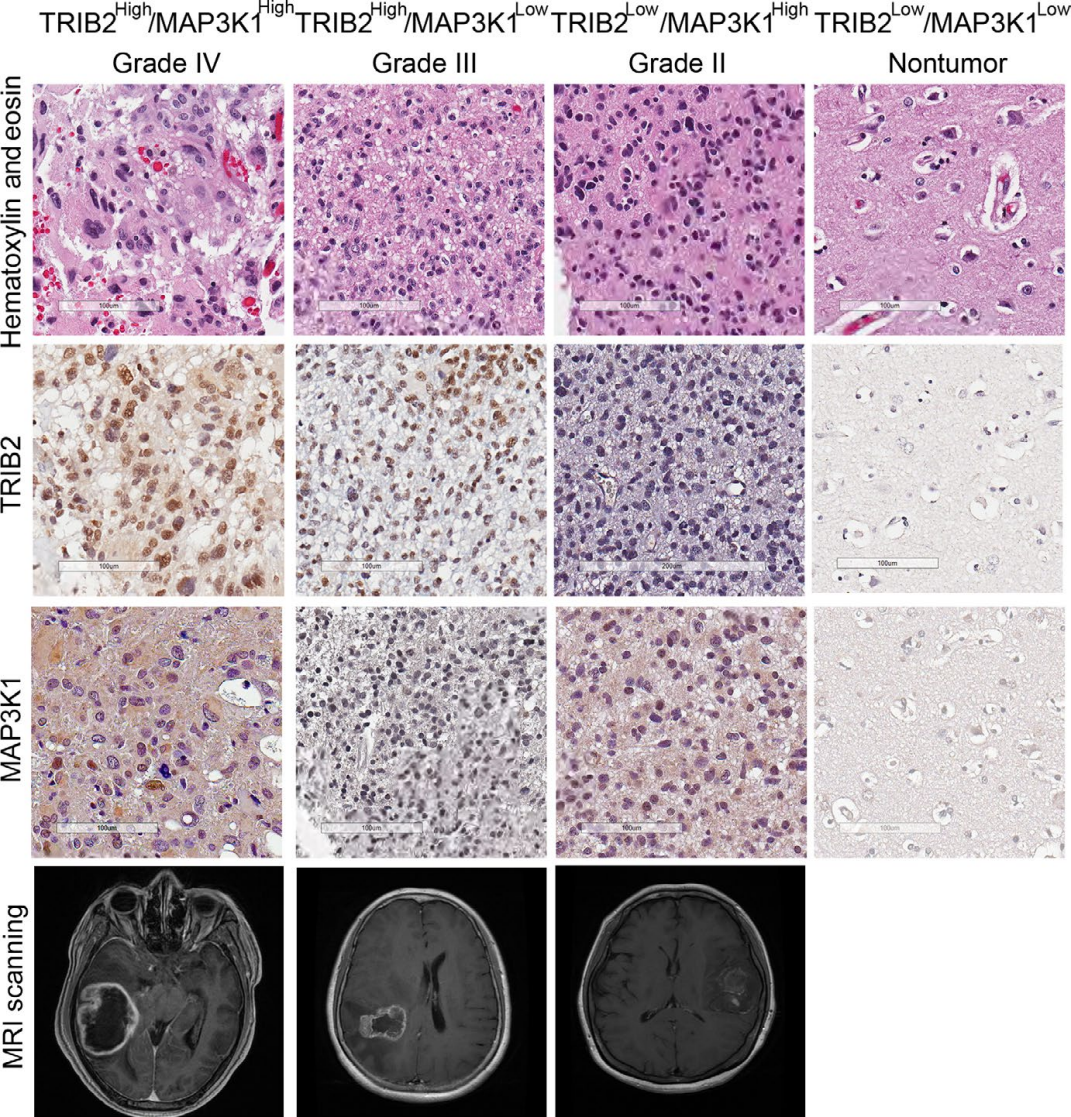
Representative IHC images of TRIB2 and MAP3K1 in glioma samples. Upper panel: H&E staining; second panel: TRIB2 staining; third panel: MAP3K1 staining; lower panel: presurgical dynamic contrast‐enhanced scanning. Brain tissue from epilepsy surgery was used as a negative control

**Figure 6:**
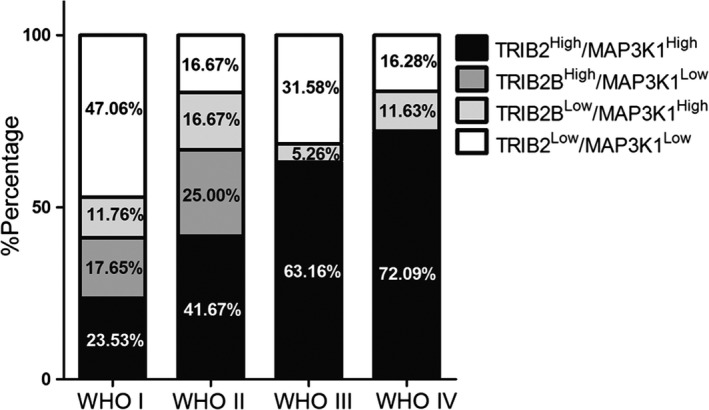
TRIB2 and MAP3K1 were enriched in high‐grade glioma samples. TRIB2^High^/MAP3K1High samples accounted for 72.09% in GBM and 63.16% in grade III gliomas compared with the grade II and grade I glioma (41.67% for grade II and 23.53% for grade I gliomas)

IHC was performed on 91 glioma samples to analyze the expression of TRIB2 and MAP3K1, and three brain tissues from epilepsy surgery were used as negative controls. The baseline information for the patients is shown in Table 1. The IHC results showed that TRIB2 was predominantly expressed in the nucleus, and MAP3K1 was enriched in the cytoplasm and cytomembrane of glioma cells, while no significant staining was observed in the nontumor tissues (Figure 5). In addition, the patients with glioma were divided into two groups according to the GIS of TRIB2 and MAP3K1 expression. The data indicated that TRIB2 and MAP3K1 were likely to be enriched in GBM and grade III glioma (TRIB2^High^/MAP3K1High samples accounted for 72.09% in GBM and 63.16% in grade III glioma) compared with grade I and grade II glioma (Figure 6). Additionally, TRIB2 and MAP3K1 overexpression was associated with the histological grade of glioma (*χ* ^2^ = 18.86, *P* < 0.01 for TRIB2 and* χ* ^2^ = 38.06, *P* < 0.01 for MAP3K1, Table 2). Moreover, coexpression of TRIB2 and MAP3K1 (TRIB2^High^/MAP3K1High) was identified in 52 of all 91 samples (57.14%) and showed statistical significance (Pearson's* χ* ^2^ = 34.12, *P* < 0.01), indicating that high expression of TRIB2 and MAP3K1 was markedly associated with the histological classification in glioma (Table 3). However, no significant association was observed when comparing TRIB2 or MAP3K1 expression with other clinicopathological features, including age, sex, tumor location, and presurgical epilepsy (Table 2).

**Table 1:** Baseline information for glioma patients involved in this study

Patient characteristics	Patient number
Total	91
Age	45.56 ± 15.65
Gender	
Male	43
Female	48
WHO grade	
I	20
II	19
III	15
IV	37
Postsurgical radiotherapy	
Yes	78
No	13
Postsurgical TMZ therapy	
Yes	80
No	11
Tumor location	
Frontal lobe	22
Temporal lobe	34
Occipital lobe	12
Parietal lobe	16
Cerebellum	7
Presurgical epilepsy	
Yes	23
No	68

**Table 2:** Association of TRIB2 and MAP3K1 expression with clinicopathological parameters in glioma patients

	n (%)	TRIB2 expression				MAP3K1 Expression			
		High	Low	*χ* ^2^	*P*	High	Low	*χ* ^2^	*P*
Age				0.022	0.882			1.738	0.187
<50	57 (62.64%)	36	21			36	21		
>50	34 (37.36%)	22	12			26	8		
Gender				1.282	0.257			0.019	0.894
Male	43 (47.25%)	30	13			29	14		
Female	48 (52.75%)	28	20			33	15		
WHO grade				18.863	1.4e‐10*			38.064	6.84e‐10*
I‐II	39 (31.87%)	15	14			13	16		
III‐IV	52 (68.13%)	43	19			49	13		
Location				4.451	0.348			1.246	0.870
Occipital lobe	12 (13.19%)	5	7			8	4		
Parietal lobe	16 (17.58%)	11	5			10	6		
Temporal lobe	34 (37.36%)	25	9			23	11		
Frontal lobe	22 (24.18%)	13	9			15	7		
Cerebellum	7 (7.69%)	4	3			6	1		
Presurgical epilepsy				0.109	0.741			0.748	0.387
Yes	23 (25.27%)	14	9			14	9		
No	68 (74.73%)	44	24			48	20		

* *P* < 0.01

**Table 3:** Combination of TRIB2 and MAP3K1 expression in glioma patients

MAP3K1 expression (%)	TRIB2 expression (%)		Pearson *χ* ^2^	*P*
	High	Low		
High	52 (57.14%)	10 (10.99%)	34.12	5.17e‐9*
Low	6 (6.59%)	23 (25.27%)		

* *P* < 0.01

### 3.5 The combined increase in TRIB2 and MAP3K1 suggested a poor prognosis of patients with glioma

**Figure 7:**
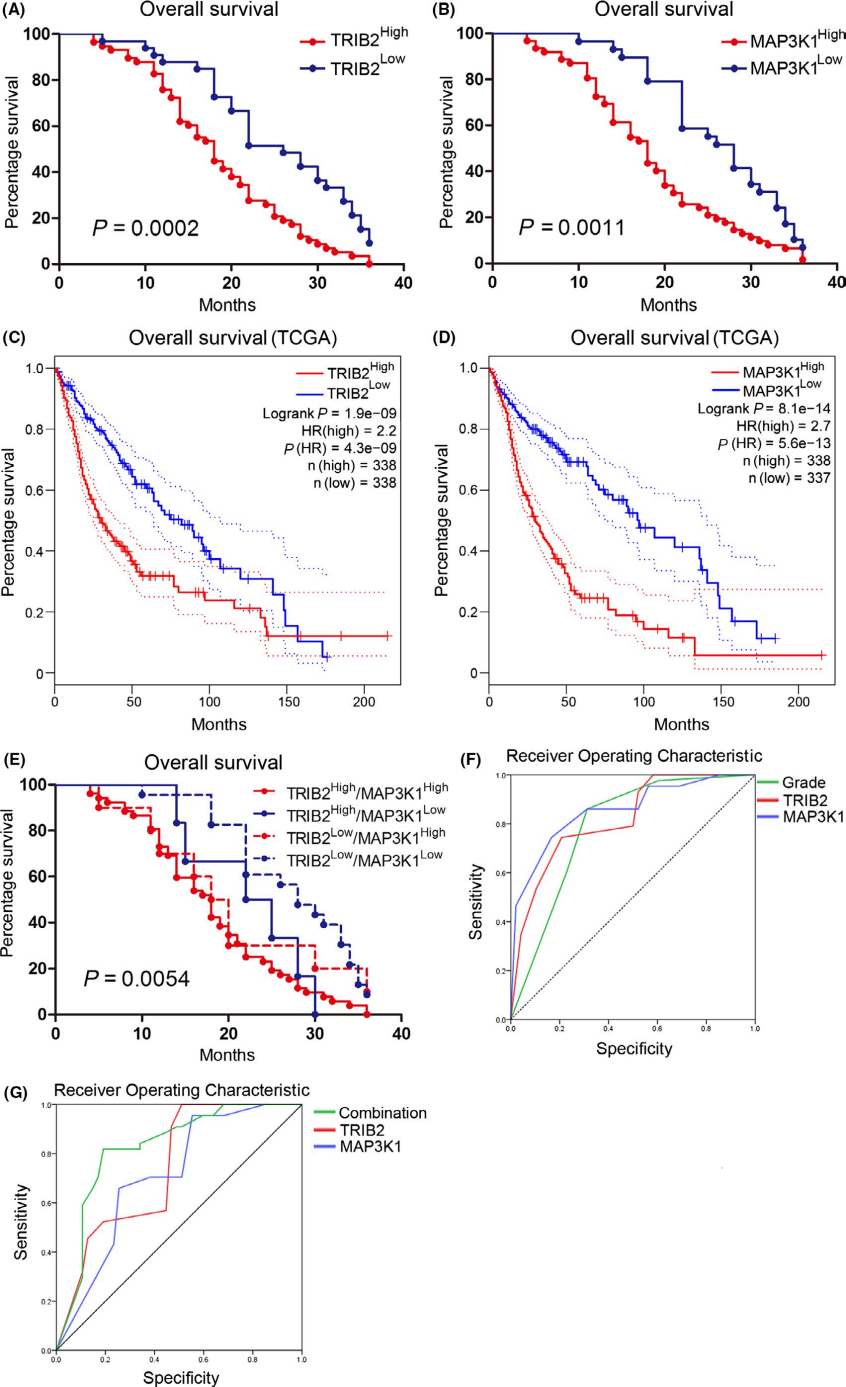
Combined elevations of TRIB2 and MAP3K1 indicated poor prognosis in glioma patients. A, Kaplan‐Meier analysis for TRIB2 expression with patient samples (*P* = 0.0002, with log‐rank test). B, Kaplan‐Meier analysis for MAP3K1 expression with patient samples (*P* = 0.0011, with log‐rank test). C, Kaplan‐Meier analysis with TCGA for TRIB2 expression (*P* < 0.01, with log‐rank test). D, Kaplan‐Meier analysis with TCGA for MAP3K1 expression (*P* < 0.01, with log‐rank test). E, Kaplan‐Meier analysis for combined expression of TRIB2 and MAP3K1 with patient samples (*P* = 0.0054, with log‐rank test). F, ROC analysis for pathological grades, TRIB2 expression, and MAP3K1 expression with overall survival in patient samples. TRIB2: AUC = 0.822, *P* < 0.01, with *z* test; MAP3K1: AUC = 0.822, *P* < 0.01, with *z* test; WHO Grade: AUC = 0.789, *P* = 0.000002, with *z* test. G, ROC analysis for combination of TRIB2 and MAP3K1 expression, TRIB2 expression and MAP3K1 expression with overall survival in patient samples. TRIB2: AUC = 0.822, *P* < 0.01, with z test; MAP3K1: AUC = 0.822,* P* < 0.01, with *z* test; combination of TRIB2 and MAP3K1 expression: AUC = 0.889, *P* = 0.000002, with *z* test

As mentioned previously, correlated overexpression of TRIB2 and MAP3K1 could be associated with high‐grade glioma. The survival of the patients was monitored until December 2016 and analyzed using the Kaplan‐Meier method according to the expression of TRIB2 and MAP3K1. We found that patients with glioma and lower expression of TRIB2 or MAP3K1 exhibited prolonged overall survival compared with patients with glioma and higher TRIB2 or MAP3K1 expression (Figure 7A,B). Similar results were observed when the Rembrandt database was used to analyze 676 patient samples (Figure 7C,D).

Furthermore, to assess the prognostic roles of the combined expression of TRIB2 and MAP3K1 in glioma, we obtained the following four combinations using 91 tumor samples according to the IHC expression levels: TRIB2^High^/MAP3K1High, TRIB2^High^/MAP3K1Low, TRIB2^Low^/MAP3K1High, and TRIB2^Low^/MAP3K1Low. The results showed that the median survival of patients with glioma in the TRIB2^High^/MAP3K1High group was 18 months compared with 28 months in the TRIB2^Low^/MAP3K1Low group. Moreover, co‐overexpression of TRIB2 and MAP3K1 (TRIB2^High^/MAP3K1High) tended to indicate shorter overall survival than other expression combinations in patients with glioma (Figure 7E and Table 4). Additionally, ROC analysis was performed to evaluate the sensitivity and specificity of TRIB2 and MAP3K1 for the prediction of outcomes in patients with glioma. The results indicated that the AUCs of TRIB2 and MAP3K1 were 0.822 (*P* < 0.01) and 0.855 (*P* < 0.01), respectively, and the AUC of the WHO classification of glioma was 0.789 (*P* = 0.000002), indicating that combined increases in TRIB2 and MAP3K1 are associated with significant sensitivity and specificity for the prediction of a poor prognosis in patients with glioma (Figure 7F and Table 5). Additionally, ROC analysis also showed an increased sensitivity and specificity for the combination of TRIB2 and MAP3K1 on the evaluation of survival compared with TRIB2 or MAP3K1 expression alone (Figure 7G and Table 5). In addition, we grouped other potentially related prognostic factors for survival analysis using the log‐rank test. The results indicated that multiple clinical characteristics, including age >50, high WHO grade, and presurgical epilepsy, were significant prognostic indicators for the survival of patients with glioma (Figure S1A,B,C, and Table 4). Although gene expression alterations associated with cancer survival were thoroughly investigated, more studies are required to fully understand the underlying biological mechanisms.

**Table 4:** Univariate analysis of survival data

Clinical characteristics	*P* Value
Age, <50 y compared to >50 y	<0.0001**
Gender, male compared to female	0.536
WHO grade, I‐II compared to III‐IV	<0.0001**
Location, compared among tumors with different locations	0.1031
Epilepsy, presurgical epilepsy compared to nonepilepsy	0.0494*
TRIB2 expression, high compared to Low	0.0002**
MAP3K1 expression, high compared to Low	0.0001**
Coexpression of TRIB2 and MAP3K1, TRIB2^High^/MAP3K1High compared to others	0.0054**

* *P* < 0.05

** *P* < 0.01

**Table 5:** ROC analysis for predict factors in gliomas

Variables	AUC	*P* Value
WHO grade	0.789	0.000002*
TRIB2 expression	0.820	1.4831e‐7*
MAP3K1 expression	0.855	5.7788e‐9*
Combination of TRIB2 and MAP3K1	0.889	9.4539E‐8*

* *P* < 0.01.

### 3.6 Enriched TRIB2 and MAP3K1 were associated with TMZ resistance and radioresistance in glioma

**Figure 8:**
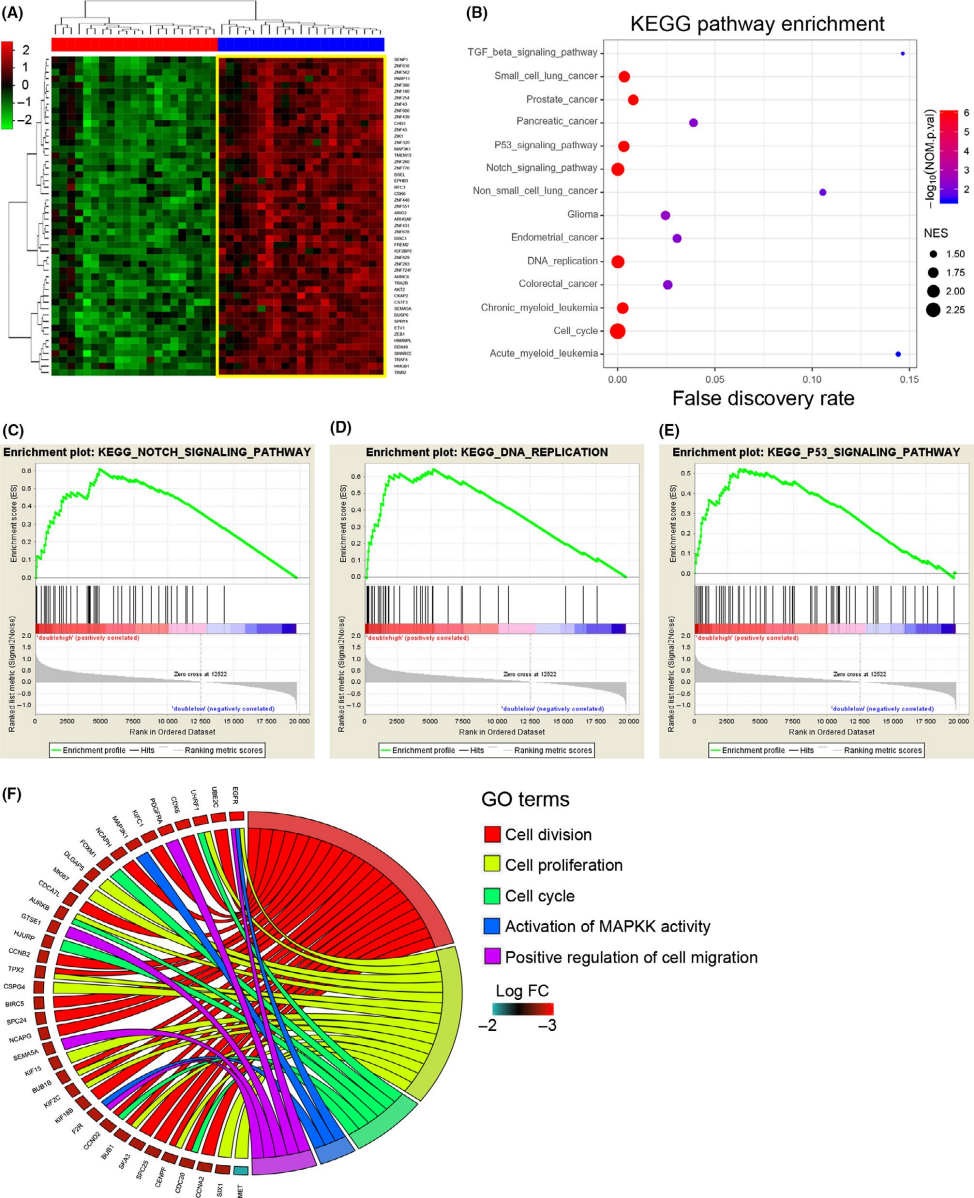
Combined elevation of TRIB2 and MAP3K1 was correlated to multiple pathways which were associated with tumorigenesis and therapy resistance. A, Hierarchical bi‐clustering analysis with TCGA database indicated that significant gene signatures in TRIB2^High^/MAP3K1High GBM compared with TRIB2^Low^/MAP3K1Low GBM. B, KEGG pathway analysis with TCGA database for TRIB2^High^/MAP3K1High GBM compared with TRIB2^Low^/MAP3K1Low GBM. C‐E, GSEA analysis with TCGA database for TRIB2^High^/MAP3K1High GBM compared with TRIB2^Low^/MAP3K1Low GBM. The results demonstrated multiple correlated pathways including NOTCH pathway (C), DNA replications (D), and p53 pathway (E). F, GO analysis with TCGA database for TRIB2^High^/MAP3K1High GBM compared with TRIB2^Low^/MAP3K1Low GBM

As described previously, our kinome‐wide screening results identified that TIRB2 was correlated with pathological malignancy and resistance to radiotherapy and TMZ in glioma. The TCGA database was grouped into two groups according to the expression of TRIB2 and MAP3K1 to investigate the potential biological function of TRIB2 and MAP3K1 in GBM. Hierarchical bi‐clustering analysis indicated significant gene signatures in TRIB2^High^/MAP3K1High GBM compared withTRIB2^Low^/MAP3K1Low GBM (Figure 8A). Additionally, KEGG pathway analysis demonstrated multiple molecular pathways that were related to malignant cancer and therapeutic resistance, including cell cycle pathways^24^ and the NOTCH,^25^ DNA replication,^26^ and p53^27^ pathways. Similar results were observed by gene set enrichment analysis (GSEA) (Figure 8 C,D, and E). Detailed data for KEGG and GSEA are shown in Figures S2 and S3. Moreover, GO analysis also showed a strong correlation between the TRIB2^High^/MAP3K1High gene signature and multiple pathways associated with malignant cancers (Figure 8F). Interestingly, activation of MAPKK activity was also confirmed to be regulated by aberrant activation of TRIB2 (Figure 8F).

**Figure 9:**
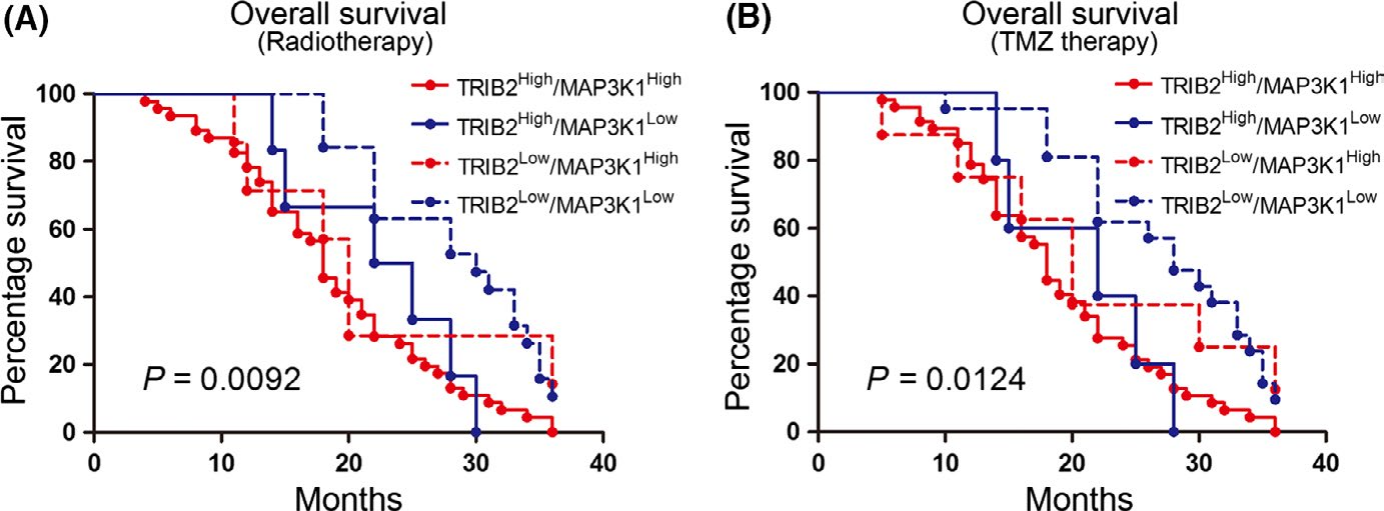
Enriched TRIB2 and MAP3K1 were associated with TMZ and radioresistance in glioma. A, Kaplan‐Meier analysis for combined expression of TRIB2 and MAP3K1 in glioma patients with postsurgical radiotherapy (*P* = 0.0092, with log‐rank test). B, Kaplan‐Meier analysis for combined expression of TRIB2 and MAP3K1 in glioma patients received postsurgical TMZ therapy (*P* = 0.0124, with log‐rank test)

To clarify the potential predictive biomarkers of therapeutic resistance and pathological characteristics, the antitherapeutic characteristics of TRIB2 and MAP3K1 were analyzed. Survival data of 78 patients who received radiotherapy were extracted, and the Kaplan‐Meier analysis showed that high expression of TRIB2 and MAP3K1 was conversely correlated with the poor survival of patients with glioma who were treated with radiotherapy (Figure 9A). Similar results were observed for patients who received TMZ therapy (Figure 9B). Overall, these data suggest that enriched TRIB2 and MAP3K1, which could be used to predict the efficacy of routine adjuvant treatment for glioma, may indicate increased resistance to TMZ and radiotherapy.

## 4 DISCUSSION

Glioma accounts for 46% of all primary intracranial tumors in the central nervous system.^28^ Increasing evidence has suggested that rapid acquired therapeutic resistance is the major cause of the failure of standard comprehensive treatments, leading to the recurrence and high mortality of glioma.^29^, ^30^ Various molecular pathways have been shown to be responsible for the acquisition of therapeutic resistance in glioma.^26^, ^31^, ^32^, ^33^ Therefore, it is essential to identify the mechanisms, especially prognostic biomarkers, underlying therapeutic resistance in glioma. In this study, we analyzed the transcriptome expression profiles of 3 published GEO databases related to pathological classification, radioresistance, and TMZ resistance and identified TRIB2 as the most correlated kinase‐encoding gene that induces malignant phenotypes, such as higher pathological grade, radioresistance, and TMZ resistance in glioma. Additionally, MAP3K1 was identified as the most correlated gene to TRIB2. Next, combined overexpression of TRIB2 and MAP3K1 was identified in glioma and correlated with a poor prognosis of glioma with significant sensitivity and specificity. Moreover, enriched TRIB2 and MAP3K1 indicated resistance to TMZ and radiotherapy. Overall, our findings suggest that the combined increase in TRIB2 and MAP3K1 could be prognostic biomarkers and potential therapeutic targets for glioma.

Abnormally elevated TRIB2 was previously identified as the clinically relevant factor in various subtypes of human cancers.^5^, ^6^, ^7^, ^8^ As described previously, TRIB2 promotes tumorigenesis and induces therapeutic resistance by activating Wnt‐mediated downstream activation of Hippo signaling and the YAP pathway through transcriptional control of gene expression and TRIB2‐mediated posttranslational regulation of protein stability in liver cancer cells.^34^ Richard et al^10^ indicated that TRIB2 enhances the tumorigenesis of melanoma cells by negatively regulating p21 and p53 in an AKT serine/threonine kinase‐dependent manner. In addition to the analysis of published gene profiles, we also validated our findings using clinical samples obtained from patients with glioma. Importantly, a strong correlation between TRIB2 expression and survival and therapeutic response to chemotherapeutic and radiotherapeutic resistance was observed. However, the underlying mechanisms for the glioma‐promoting functions of TRIB2 require further investigation.

MAP3K1 is a 196‐kDa serine/threonine kinase that is activated by various stimuli and cell stresses, including growth factors, cytokines, and microtubule disruption.^35^ Our present study showed elevated MAP3K1 expression in glioma, which is conversely associated with a poor prognosis and therapeutic resistance in glioma. Similar to our study, recent studies have shown that MAP3K1 is functionally required for multiple physiological processes, including cell growth, cell migration, and apoptosis, and is strongly correlated with poor outcomes in a wide range of malignant cancers.^13^, ^14^, ^15^ Furthermore, another study demonstrated that MAP3K1 promotes tumorigenesis in nonsmall cell lung cancer cells by regulating epithelial‐mesenchymal transition through miR‐145‐5p‐mediated JNK signaling pathway activation, suggesting the potential role of MAP3K1 as an important oncogene.^12^, ^15^, ^35^

Increasing evidence has indicated that acquired resistance to chemotherapy and radiation leads to the rapid recurrence and poor prognosis of glioma in patients.^36^ According to our results, MAP3K1 was the gene that was most correlated with TRIB2. Moreover, elevated coexpression of TRIB2 and MAP3K1 was significantly correlated with a poor prognosis and indicated therapeutic resistance in glioma. These novel findings indicated that TRIB2 and MAP3K1 could be potential predictors for evaluating the survival and efficacy of routine adjuvant treatments of glioma. In addition, multiple clinical characteristics, including age, pathological classification, and presurgical epilepsy, were significant prognostic indicators for the survival of patients with glioma. Interestingly, our survival analysis showed that patients with glioma and increased expression of TRIB2 and MAP3K1 exhibited worse responses to TMZ and radiotherapy. The ROC analysis confirmed that the sensitivity and specificity of TRIB2/MAP3K1 expression were statistically significant, indicating that TRIB2/MAP3K1 expression should be investigated for evaluating the prognosis and therapeutic efficacy in patients with glioma. These two molecules could be important biomarkers for traditional pathological classification in glioma. Overall, this study demonstrated the potential of TRIB2 and MAP3K1 as therapeutic targets in glioma.

## 5 CONCLUSION

TRIB2 and MAP3K1 could be used as novel therapeutic targets and prognostic biomarkers to evaluate the malignancy and long‐term outcome of glioma.

## CONFLICT OF INTEREST

The authors declare no conflict of interest.

## References

[1] Cui H , Zhang M , Wang Y , Wang Y . NF‐YC in glioma cell proliferation and tumor growth and its role as an independent predictor of patient survival. Neurosci Lett. 2016;631:40‐49. 27495011 10.1016/j.neulet.2016.08.003

[2] Nie S , Chen T , Yang X , Huai P , Lu M . Association of Helicobacter pylori infection with esophageal adenocarcinoma and squamous cell carcinoma: a meta‐analysis. Dis Esophagus. 2014;27(7):645‐653. 24635571 10.1111/dote.12194

[3] Wang T , Niu X , Gao T , et al. Prognostic Factors for Survival Outcome of High‐Grade Multicentric Glioma. World Neurosurg. 2018;112:e269‐e277. 29337167 10.1016/j.wneu.2018.01.035

[4] Kim Y‐W , Liu TJ , Koul D , et al. Identification of novel synergistic targets for rational drug combinations with PI3 kinase inhibitors using siRNA synthetic lethality screening against GBM. Neuro Oncol. 2011;13(4):367‐375. 21430111 10.1093/neuonc/nor012 PMC3064700

[5] Liang Y , Yu D , Perez‐Soler R , Klostergaard J , Zou Y . TRIB2 contributes to cisplatin resistance in small cell lung cancer. Oncotarget. 2017;8(65):109596‐109608. 29312632 10.18632/oncotarget.22741 PMC5752545

[6] Yao B , Xu Y , Wang J , et al. Reciprocal regulation between O‐GlcNAcylation and tribbles pseudokinase 2 (TRIB2) maintains transformative phenotypes in liver cancer cells. Cell Signal. 2016;28(11):1703‐1712. 27515988 10.1016/j.cellsig.2016.08.003

[7] Hou Z , Guo K , Sun X , et al. TRIB2 functions as novel oncogene in colorectal cancer by blocking cellular senescence through AP4/p21 signaling. Mol Cancer. 2018;17(1):172. 30541550 10.1186/s12943-018-0922-x PMC6291992

[8] Foulkes DM , Byrne DP , Yeung W , et al. Covalent inhibitors of EGFR family protein kinases induce degradation of human Tribbles 2 (TRIB2) pseudokinase in cancer cells. Sci Signal. 2018;11(549):eaat7951. 30254057 10.1126/scisignal.aat7951 PMC6553640

[9] Keeshan K , He Y , Wouters BJ , et al. Tribbles homolog 2 inactivates C/EBPalpha and causes acute myelogenous leukemia. Cancer Cell. 2006;10(5):401‐411. 17097562 10.1016/j.ccr.2006.09.012 PMC2839500

[10] Hill R , Madureira PA , Ferreira B , et al. TRIB2 confers resistance to anti‐cancer therapy by activating the serine/threonine protein kinase AKT. Nat Commun. 2017;8:14687. 28276427 10.1038/ncomms14687 PMC5347136

[11] Uhlik MT , Abell AN , Cuevas BD , Nakamura K , Johnson GL . Wiring diagrams of MAPK regulation by MEKK1, 2, and 3. Biochem Cell Biol. 2004;82(6):658‐663. 15674433 10.1139/o04-114

[12] Cuevas BD , Abell AN , Johnson GL . Role of mitogen‐activated protein kinase kinase kinases in signal integration. Oncogene. 2007;26(22):3159‐3171. 17496913 10.1038/sj.onc.1210409

[13] Wu L , Yin JH , Guan YY , et al. A long noncoding RNA MAP3K1‐2 promotes proliferation and invasion in gastric cancer. Onco Targets Ther. 2018;11:4631‐4639. 30122954 10.2147/OTT.S168819 PMC6086095

[14] Liu C , Wang S , Zhu S , et al. MAP3K1‐targeting therapeutic artificial miRNA suppresses the growth and invasion of breast cancer in vivo and in vitro. Springerplus. 2016;5:11. 26759750 10.1186/s40064-015-1597-z PMC4700027

[15] Chang Y , Yan W , Sun C , Liu Q , Wang J , Wang M . miR‐145‐5p inhibits epithelial‐mesenchymal transition via the JNK signaling pathway by targeting MAP3K1 in non‐small cell lung cancer cells. Oncol Lett. 2017;14(6):6923‐6928. 29344125 10.3892/ol.2017.7092 PMC5754887

[16] Pham TT , Angus SP , Johnson GL . MAP3K1: Genomic Alterations in Cancer and Function in Promoting Cell Survival or Apoptosis. Genes Cancer. 2013;4(11–12):419‐426. 24386504 10.1177/1947601913513950 PMC3877667

[17] Zhen Y , Lee IJ , Finkelman FD , Shao WH . Targeted inhibition of Axl receptor tyrosine kinase ameliorates anti‐GBM‐induced lupus‐like nephritis. J Autoimmun. 2018;93:37‐44. 29895432 10.1016/j.jaut.2018.06.001 PMC6108947

[18] Pacaud R , Cheray M , Nadaradjane A , Vallette FM , Cartron PF . Histone H3 phosphorylation in GBM: a new rational to guide the use of kinase inhibitors in anti‐GBM therapy. Theranostics. 2015;5(1):12‐22. 25553095 10.7150/thno.8799 PMC4265745

[19] Bajetto A , Porcile C , Pattarozzi A , et al. Differential role of EGF and BFGF in human GBM‐TIC proliferation: relationship to EGFR‐tyrosine kinase inhibitor sensibility. J Biol Regul Homeost Agents. 2013;27(1):143‐154. 23489694

[20] Mao P , Joshi K , Li J , et al. Mesenchymal glioma stem cells are maintained by activated glycolytic metabolism involving aldehyde dehydrogenase 1A3. Proc Natl Acad Sci USA. 2013;110(21):8644‐8649. 23650391 10.1073/pnas.1221478110 PMC3666732

[21] Tso JL , Yang S , Menjivar JC , et al. Bone morphogenetic protein 7 sensitizes O6‐methylguanine methyltransferase expressing‐glioblastoma stem cells to clinically relevant dose of temozolomide. Mol Cancer. 2015;14:189. 26546412 10.1186/s12943-015-0459-1 PMC4636799

[22] Wang J , Cheng P , Pavlyukov MS , et al. Targeting NEK2 attenuates glioblastoma growth and radioresistance by destabilizing histone methyltransferase EZH2. J Clin Invest. 2017;127(8):3075‐3089. 28737508 10.1172/JCI89092 PMC5531394

[23] Velpurisiva P , Piel BP , Lepine J , Rai P . GSK461364A, a Polo‐Like Kinase‐1 Inhibitor Encapsulated in Polymeric Nanoparticles for the Treatment of Glioblastoma Multiforme. (GBM). Bioengineering (Basel). 2018;5(4):83. 10.3390/bioengineering5040083 PMC6315921 30304810

[24] Li X , Ding R , Han Z , Ma Z , Wang Y . Targeting of cell cycle and let‐7a/STAT3 pathway by niclosamide inhibits proliferation, migration and invasion in oral squamous cell carcinoma cells. Biomed Pharmacother. 2017;96:434‐442. 29031202 10.1016/j.biopha.2017.09.149

[25] Majidinia M , Alizadeh E , Yousefi B , Akbarzadeh M , Zarghami N . Downregulation of Notch Signaling Pathway as an Effective Chemosensitizer for Cancer Treatment. Drug Res (Stuttg). 2016;66(11):571‐579. 27701712 10.1055/s-0042-111821

[26] Bélanger F , Fortier E , Dubé M , et al. Replication protein A availability during DNA replication stress is a major determinant of cisplatin resistance in ovarian cancer cells. Cancer Res. 2018;78(19):5561‐5573. 30072396 10.1158/0008-5472.CAN-18-0618

[27] Nie ER , Jin X , Wu W , et al. BACH1 promotes temozolomide resistance in glioblastoma through antagonizing the function of p53. Sci Rep. 2016;6:39743. 28000777 10.1038/srep39743 PMC5175153

[28] Asklund T , Malmstrom A , Bergqvist M , Bjor O , Henriksson R . Brain tumors in Sweden: data from a population‐based registry 1999–2012. Acta Oncol. 2015;54(3):377‐384. 25383446 10.3109/0284186X.2014.975369

[29] Osuka S , Van Meir EG . Overcoming therapeutic resistance in glioblastoma: the way forward. J Clin Invest. 2017;127(2):415-426. 28145904 10.1172/JCI89587 PMC5272196

[30] Saki M , Makino H , Javvadi P , et al. EGFR Mutations Compromise Hypoxia‐Associated Radiation Resistance through Impaired Replication Fork‐Associated DNA Damage Repair. Mol Cancer Res. 2017;15(11):1503‐1516. 28801308 10.1158/1541-7786.MCR-17-0136 PMC5668182

[31] Tan C , Liu L , Liu X , et al. Activation of PTGS2/NF‐kappaB signaling pathway enhances radiation resistance of glioma. Cancer Med. 2019;8(3):1175‐1185. 30740906 10.1002/cam4.1971 PMC6434213

[32] Wang Y , Xu H , Liu T , et al. Temporal DNA‐PK activation drives genomic instability and therapy resistance in glioma stem cells. JCI Insight. 2018;3(3):e98096. 10.1172/jci.insight.98096 PMC5821187 29415883

[33] Arezi B , McKinney N , Hansen C , et al. Compartmentalized self‐replication under fast PCR cycling conditions yields Taq DNA polymerase mutants with increased DNA‐binding affinity and blood resistance. Front Microbiol. 2014;5:408. 25177317 10.3389/fmicb.2014.00408 PMC4132270

[34] Wang J , Park JS , Wei Y , et al. TRIB2 acts downstream of Wnt/TCF in liver cancer cells to regulate YAP and C/EBPalpha function. Mol Cell. 2013;51(2):211‐225. 23769673 10.1016/j.molcel.2013.05.013 PMC4007693

[35] Avivar‐Valderas A , McEwen R , Taheri‐Ghahfarokhi A , et al. Functional significance of co‐occurring mutations in PIK3CA and MAP3K1 in breast cancer. Oncotarget. 2018;9(30):21444‐21458. 29765551 10.18632/oncotarget.25118 PMC5940413

[36] Gallego‐Perez D , Chang L , Shi J , et al. On‐Chip Clonal Analysis of Glioma‐Stem‐Cell Motility and Therapy Resistance. Nano Lett. 2016;16(9):5326‐5332. 27420544 10.1021/acs.nanolett.6b00902 PMC5040341

