# Supplementary figures and images for: Combined elevation of TRIB2 and MAP3K1 indicates poor prognosis and chemoresistance to temozolomide in glioblastoma
Source: CNS Neurosci Ther. 2019 Jul 18;26(3):297–308. doi: 10.1111/cns.13197 (PMC7053231; doi:10.1111/cns.13197)

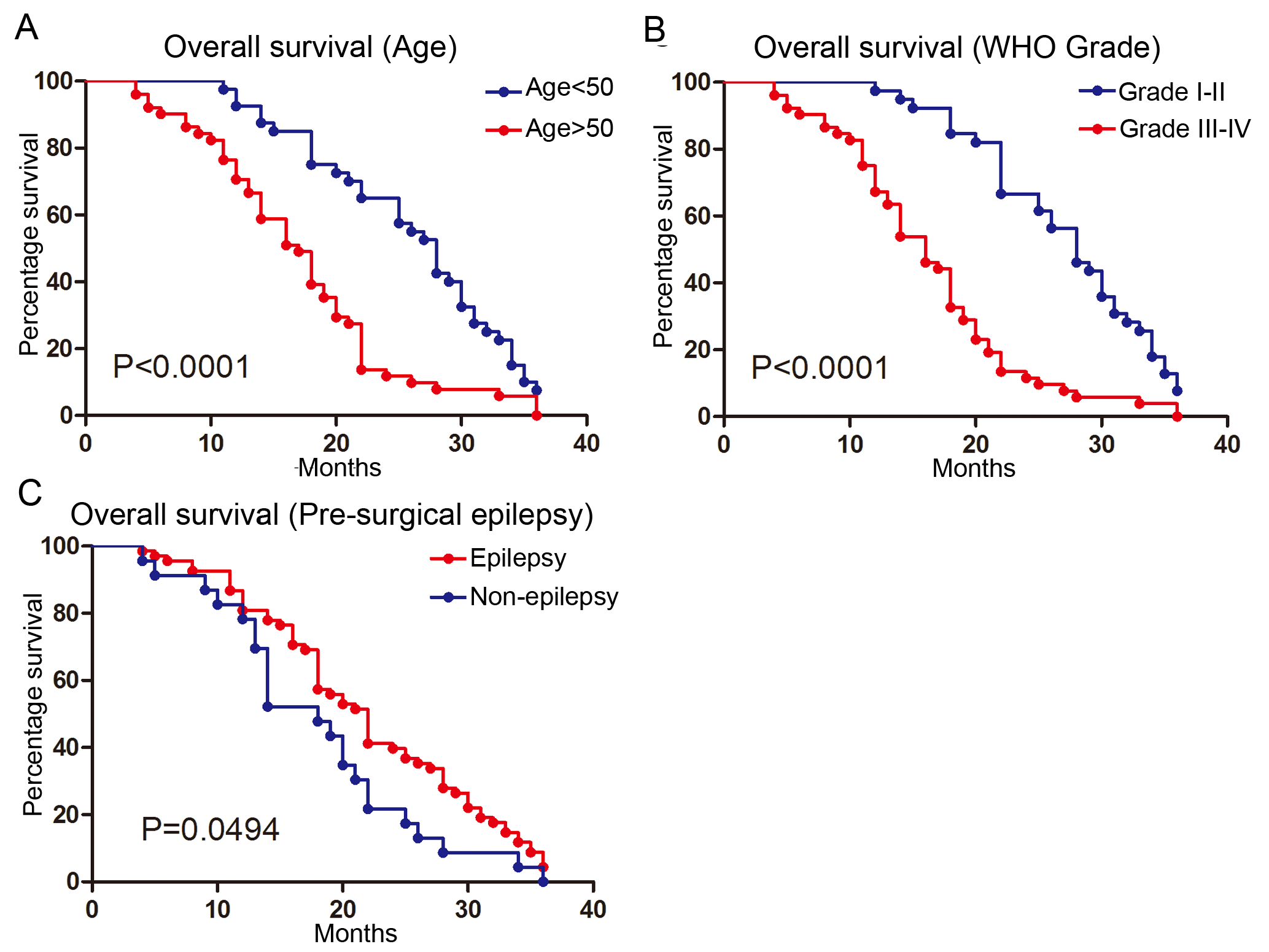

Supplement: Supplementary file 1 [file CNS-26-297-s001.tif]

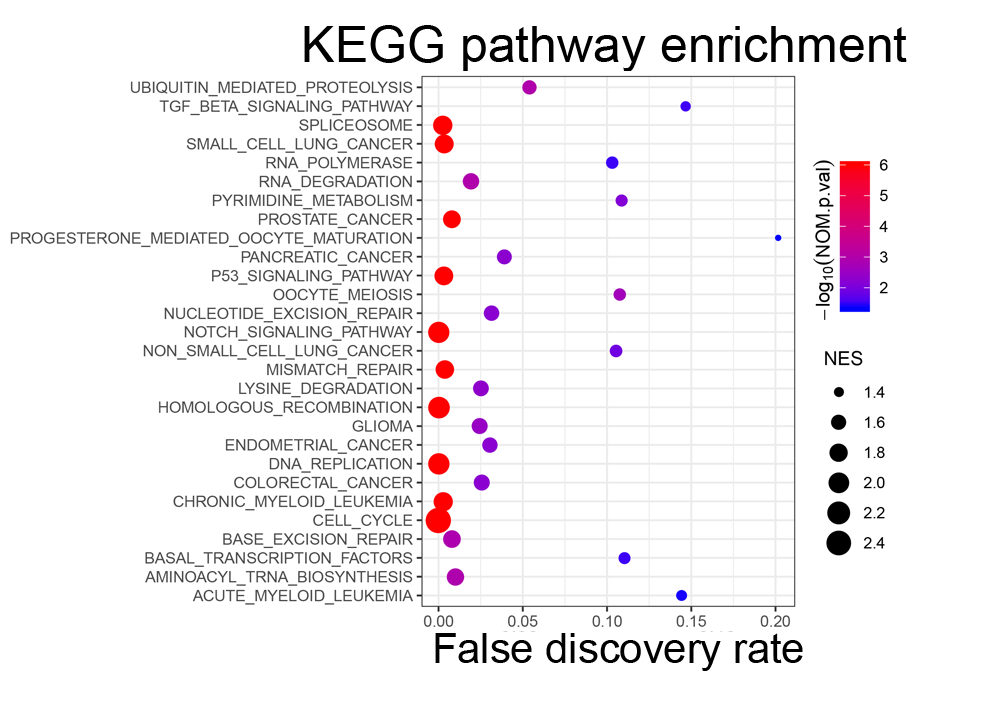

Supplement: Supplementary file 2 [file CNS-26-297-s002.tif]

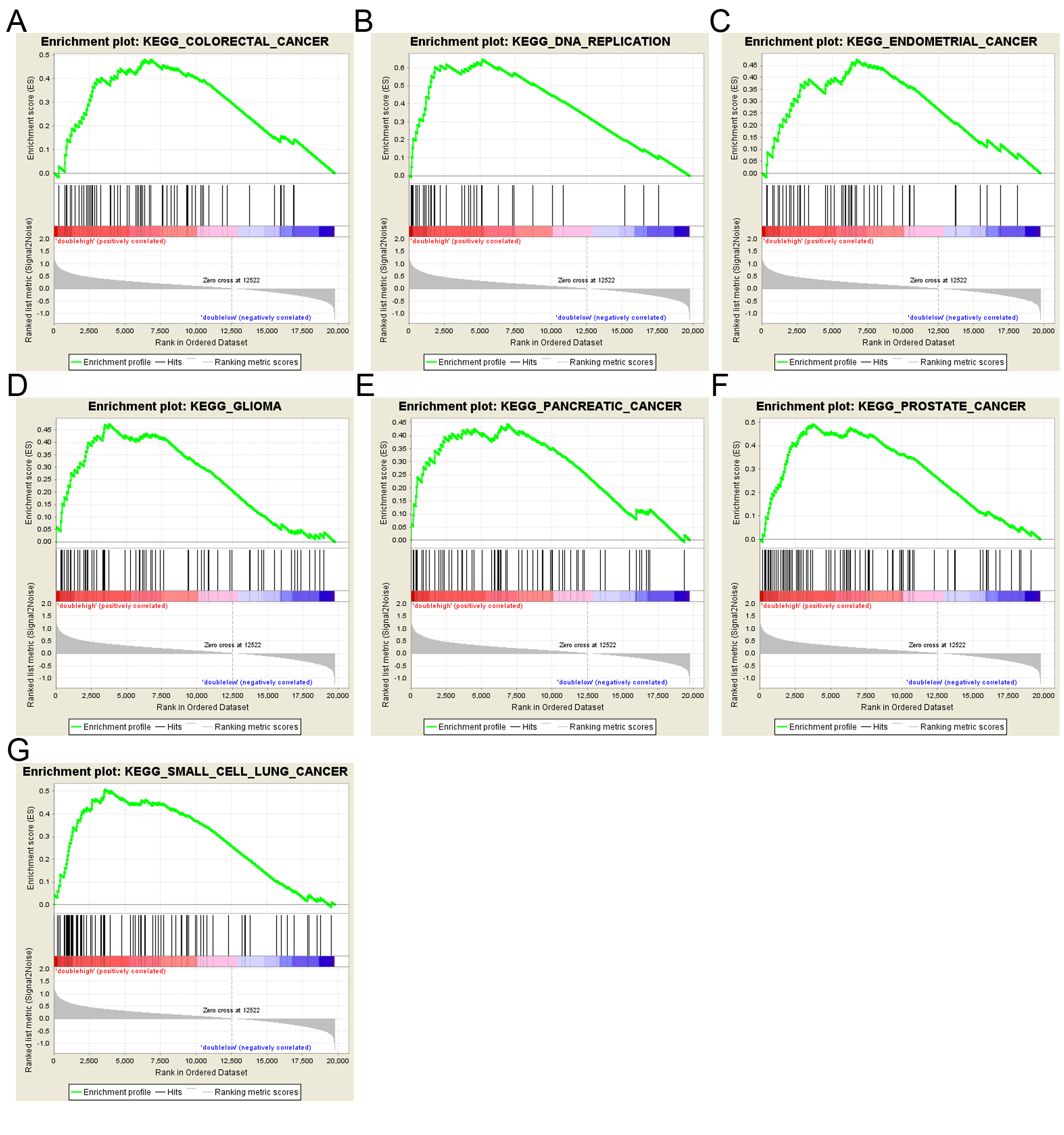

Supplement: Supplementary file 3 [file CNS-26-297-s003.tif]
